# Supplementary material for: Nicotine and Its Downstream Metabolites in Maternal and Cord Sera: Biomarkers of Prenatal Smoking Exposure Associated with Offspring DNA Methylation
Source: Int J Environ Res Public Health. 2020 Dec 20;17(24):9552. doi: 10.3390/ijerph17249552 (PMC7766890; doi:10.3390/ijerph17249552)
Supplement: Supplementary file 1 [file ijerph-17-09552-s001.zip › supplementary/Supplementary figure 1 a.docx]

Supplementary Figure 2 (a). Pairwise scatter plot of nicotine and its metabolites levels in maternal sera (F0 mothers of F1). Dots with the same color and shape belong to the same cluster (K-means cluster analysis). For the five clusters of F0-metabolites, the red colored dots represent the cluster with lowest levels of metabolites (non-exposed cluster).
