# Supplementary material for: Nicotine and Its Downstream Metabolites in Maternal and Cord Sera: Biomarkers of Prenatal Smoking Exposure Associated with Offspring DNA Methylation
Source: Int J Environ Res Public Health. 2020 Dec 20;17(24):9552. doi: 10.3390/ijerph17249552 (PMC7766890; doi:10.3390/ijerph17249552)
Supplement: Supplementary file 1 [file ijerph-17-09552-s001.zip › supplementary/Supplementary figure 2.pdf]

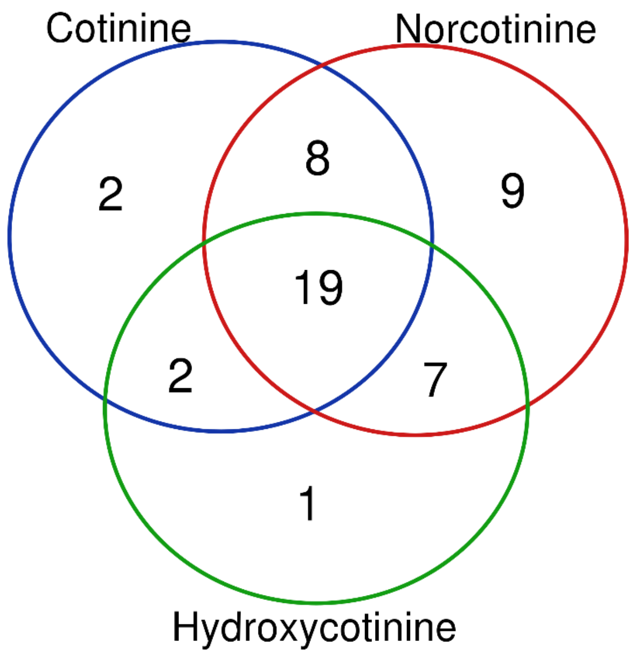

Nicotine metabolites	Number of overlapping CpGs	Overlapping CpG names
Cotinine Hydroxycotinine Norcotinine	19	cg18694169 cg05575921 cg05549655 cg19089201 cg04180046 cg01952185 cg12876356 cg11924019 cg14179389 cg06012804 cg09935388 cg12101586 cg12803068 cg15507334 cg26516004 cg13570656 cg07339236 cg21251018 cg22549041
Cotinine Norcotinine	8	cg00029284 cg10980495 cg18183624 cg18092474 cg19648306 cg07810039 cg17384889 cg10253847
Cotinine Hydroxycotinine	2	cg18473733 cg14807565
Hydroxycotinine Norcotinine	7	cg01359532 cg26652413 cg04535902 cg18146737 cg18703066 cg21611682 cg26954197
Cotinine	2	cg23553912 cg16459265
Norcotinine	9	cg08328513 cg18316974 cg18182566 cg25464840 cg27434149 cg16449012 cg09662411 cg06501366 cg14251267
Hydroxycotinine	1	cg02063817

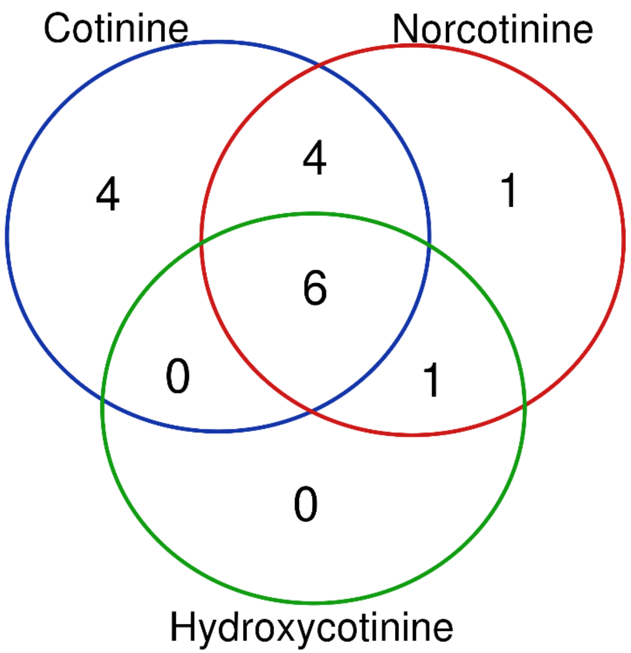

Nicotine metabolites	Number of overlapping CpGs	Overlapping CpG names
Cotinine Hydroxycotinine Norcotinine	6	cg12876356 cg09935388 cg18316974 cg18146737 cg06338710 cg09662411
Cotinine Norcotinine	4	cg15959363 cg13834112 cg05575921 cg04180046
Cotinine Hydroxycotinine	1	cg04535902
Hydroxycotinine Norcotinine	4	cg26652413 cg19089201 cg12803068 cg14179389
Cotinine	1	cg10399789
Norcotinine	6	cg12876356 cg09935388 cg18316974 cg18146737 cg06338710 cg09662411
Hydroxycotinine	4	cg15959363 cg13834112 cg05575921 cg04180046
