# Supplementary material for: Nicotine and Its Downstream Metabolites in Maternal and Cord Sera: Biomarkers of Prenatal Smoking Exposure Associated with Offspring DNA Methylation
Source: Int J Environ Res Public Health. 2020 Dec 20;17(24):9552. doi: 10.3390/ijerph17249552 (PMC7766890; doi:10.3390/ijerph17249552)
Supplement: Supplementary file 1 [file ijerph-17-09552-s001.zip › supplementary/supplementary material (methods section).docx]

Article

**Nicotine and its downstream metabolites in maternal and cord sera: Biomarkers of prenatal smoking exposure associated with offspring DNA methylation**

**Parnian Kheirkhah Rahimabad ^1^*, Thilani M. Anthony ^2^, A. Daniel Jones ^2^, Shakiba Eslamimehr ^1^, Nandini Mukherjee ^1^, Susan Ewart ^3^, John W. Holloway ^4^, Hasan Arshad ^5, 6, 7^, Sarah Commodore ^8^, Wilfried Karmaus ^1^**

1 Division of Epidemiology, Biostatistics, and Environmental Health, School of Public Health, University of Memphis, Memphis, TN, USA

2 Department of Biochemistry & Molecular Biology, Michigan State University, East Lansing, MI, USA

3 Department of Large Animal Clinical Sciences, Michigan State University, East Lansing, MI, USA

4 Human Development and Health, Faculty of Medicine, University of Southampton, Southampton, UK

5 Clinical and Experimental Sciences, Faculty of Medicine, University of Southampton, Southampton, UK

6 The David Hide Asthma and Allergy Research Centre, Isle of Wight, UK

7 NIHR Southampton Biomedical Research Centre, University Hospital Southampton, UK

8 Department of Environmental and Occupational Health, Indiana University, Bloomington, IN, USA

**Supplementary Data**

**Experimental Design, analysis of metabolites, nutrients, and toxins (MNTs)**

Serum samples were grouped, processed, and analyzed in random order. Each batch included analyses of multiple blanks, pooled quality control (QC) extracts, and extracts of reference serum, with blanks, pooled QC sera, and reference sera analyzed before and after each ten study serum extracts.

**Sample preparation**

Tubes of polypropylene microcentrifuge were loaded with 25 µL each of aliquots of water-soluble and organic-soluble stable isotope-labeled internal standard cocktails (Supplemental Table S1).

Supplemental Table S1. Composition of internal standard cocktails.

| **Internal standard solution A1 (water-soluble), in acetonitrile/water (9:1 v/v)** | **Internal standard solution L1 (organic-soluble), in 100% acetonitrile** |
| --- | --- |
| Succinic acid-*d*_4_, 5.0 µM | Palmitic acid-*d*_31_, 1.0 µM |
| Cotinine-*d*_3_, 1.0 µM | 1,2-dimyristoyl-*d*_54_-sn-glycero-3-phosphocholine, 2.0 µM |
| L-Valine-*d*_8_, 5.0 µM | 17β-estradiol-16,16,17-*d*_3_, 1.0 µM |
| [^13^C_3_]Caffeine, 5.0 µM |  |
| Thymine-*d*_3_, 5.0 µM |  |
| Phenylalanine-*d*_5_, 5.0 µM |  |

Each tube was loaded with an additional 150 µL of acetonitrile, followed by 20 µL of thawed blood serum. Tubes were vortexed and held on ice. To each tube, 200 µL of MTBE (methyl *tert*-butyl ether) and 400 µL of MilliQ water were added. Tubes were vortexed and centrifuged (10,000 x g, 4˚C, 15 min). Pipetters were used to collect 180 µL from the top organic layer. An additional 200-µL volume of MTBE was added to the aqueous (lower) layer. Following vortexing and centrifuging (as above), 180 µL of the upper layer was combined with the first MTBE fraction. This “nonpolar” fraction was evaporated to dryness under a stream of nitrogen gas and archived at -20˚C. The polar (lower) fraction was evaporated to dryness under vacuum using a SpeedVac without heat application, and residues were dissolved in 200 µL of acetonitrile/water (9:1 v/v) with vortexing followed by transfer to an amber glass auto-sampler vial with glass 200-µL insert. Extracts were maintained at -20˚C until analysis by LC-MS/MS.

**Mass Spectrometry Metadata**

| Mass spectrometer | Thermo Q-Exactive |
| --- | --- |
| Data acquisition mode | Full scan/All-ions-fragmentation |
| Ionization | Heated electrospray ionization |
| Polarity | Positive-ion |
| *m/z* range | 70-1050 |
| Spray voltage | 2500 V |
| Capillary temperature | 256˚ |
| Sheath gas flow rate | 47.6 |
| Auxiliary gas flow rate | 11.1 |
| Sweep gas flow rate | 2.34 |
| Auxiliary temperature | 410˚ |
| Maximum injection time | 200 ms |
| Resolution | 70000 (full MS)/35000 (AIF) |
| AGC target | 3 x 10^6^ |
| Run time | 0-12 min |
| Collision energy | stepped – 10, 30, 60 NCE |

**Chromatography metadata**

| Chromatograph | Thermo Vanquish Flex Binary pump with autosampler |
| --- | --- |
| Column | Acquity BEH Amide column (10 cm x 1.0 mm, 1.7 µm; Waters) |
| Flow rate | 0.30 mL/min |
| Column temperature | 30˚C |
| Injection volume | 5.0 µL |
| Mobile phase: solvent A | 100 mM ammonium acetate + 0.4% ammonium hydroxide (aqueous component adjusted to pH 9.0 before mixing) in acetonitrile/water (1:1 v/v) |
| Mobile phase: solvent B | 100 mM ammonium acetate + 0.04% ammonium hydroxide (aqueous component adjusted to pH 9.0 before mixing) in acetonitrile/water (9:1 v/v) |
| Gradient | 0.0-1.0 min (99% B); 7.0-10.0 min (50% B); 10.01 min (99%B); hold until 15 min. |

**Data processing metadata – Progenesis QI**

| Software | Progenesis QI (Waters) |
| --- | --- |
| Software version | 2.4.6911.27652 |
| Ionization polarity | Positive |
| Runs in this experiment | 1343 (includes blanks, QC, repeat injections, reference sera, study sera) |
| Feature detection | High resolution |
| Peak processing data format | Profile data |
| Data import filter value | Default |
| Peak picking: sensitivity | Absolute ion intensity; minimum intensity = 1000 |
| Peak picking: retention time limits | 0.5-11.0 minutes |
| Peak picking: adducts | [M+2H]^2+^; [M+H-H_2_O]^+^; [M+H]^+^; [M+NH_4_]^+^; [M+Na]^+^; [M+K]^+^; [M+H + C_2_H_7_N]^+^; [M+ACN+Na]^+^; [2M+H]^+^ |
| Peak picking: normalization | No normalization |
| Peak alignment: alignment reference file | QE_2019_09_19_TMA_MNT_003_Batch13_Polar_Pooled_QC_serum_POS_FullMS_AIF.raw |
| Files with alignment adjusted manually: | QE_2019_10_21* and QE_2019_10_22* (all vectors deleted, defaults used for alignment) |
| Peak identification parameter set: | None |
| Peak identification: databases used: | None |
| Peak identification: search criteria | No search performed in Progenesis |
| Compound filtering tags: | Removed all signals with maximum abundance < 10000 (2454 of 9712 signals remained); removed all with Max abundance highest in blanks; removed RMD > 1200 |
| Sample filtering: | Removed samples with outliers in cotinine-*d*_3_ signal. |
| Post-Progenesis data processing: software and version | Microsoft Excel for Mac (v. 16.42) |
| Post-Progenesis data processing: compound removal criteria | Removed all compounds highest in blanks or blanks with standards; removed all with RMD > 1200 ppm; salt cluster ions eluting at 6.5 and 7.2 min |
| Post-Progenesis data processing: normalization | Normalization to cotinine-*d*_3_ signal |

**Data processing metadata – Compound Discoverer**

| Software | Compound Discoverer (Thermo) |
| --- | --- |
| Software version | 3.0.0.294 |
| Search description and workflow | Untargeted Metabolomics workflow; Performs retention time alignment, unknown compound detection, and compound grouping across all samples. Predicts elemental compositions for all compounds, fills gaps across all samples, and hides chemical background (using Blank samples). Identifies compounds using mzCloud (using AIF MS2 spectra) and ChemSpider (formula or exact mass). Also performs similarity search for all compounds with AIF MS2 data using mzCloud. Applies mzLogic algorithm to rank order ChemSpider results. |
| General settings: | - Precursor Selection: Use MS(n - 1) Precursor  - Use Isotope Pattern in Precursor Reevaluation: True  - Provide Profile Spectra: Automatic  - Store Chromatograms: False |
| Spectrum Properties Filter: | - Lower RT Limit: 0  - Upper RT Limit: 0  - First Scan: 0  - Last Scan: 0  - Ignore Specified Scans: (not specified)  - Lowest Charge State: 0  - Highest Charge State: 0  - Min. Precursor Mass: 0 Da  - Max. Precursor Mass: 5000 Da  - Total Intensity Threshold: 0  - Minimum Peak Count: 1 |
| Scan event filters: | - Mass Analyzer: (not specified)  - MS Order: Any  - Activation Type: (not specified)  - Min. Collision Energy: 0  - Max. Collision Energy: 1000  - Scan Type: Any  - Polarity Mode: (not specified) |
| Peak filters: | - S/N Threshold (FT-only): 1.5 |
| Replacements for Unrecognized Properties: | - Unrecognized Charge Replacements: 1  - Unrecognized Mass Analyzer Replacements: ITMS  - Unrecognized MS Order Replacements: MS2  - Unrecognized Activation Type Replacements: CID  - Unrecognized Polarity Replacements: +  - Unrecognized MS Resolution@200 Replacements: 60000  - Unrecognized MSn Resolution@200 Replacements: 30000 |
| Align retention times |  |
| General settings: | - Alignment Model: Adaptive curve  - Alignment Fallback: Use Linear Model  - Maximum Shift [min]: 2  - Shift Reference File: True  - Mass Tolerance: 10 ppm  - Remove Outlier: True |
| Detect compounds: | General Settings:  - Mass Tolerance [ppm]: 5 ppm  - Intensity Tolerance [%]: 30  - S/N Threshold: 3  - Min. Peak Intensity: 1000000  - Ions:  [2M+ACN+H]+1  [2M+ACN+Na]+1  [2M+H]+1  [2M+K]+1  [2M+Na]+1  [2M+NH4]+1  [M+2H]+2  [M+ACN+2H]+2  [M+ACN+H]+1  [M+ACN+Na]+1  [M+DMSO+H]+1  [M+H]+1  [M+H+K]+2  [M+H+MeOH]+1  [M+H+Na]+2  [M+H+NH4]+2  [M+H-H2O]+1  [M+H-NH3]+1  [M+K]+1  [M+Na]+1  [M+NH4]+1  - Base Ions: [M+H]+1  - Min. Element Counts: C H  - Max. Element Counts: C90 H190 Br3 Cl4 K2 N10 Na2 O15 P3 S5  2. Peak Detection:  - Filter Peaks: True  - Max. Peak Width [min]: 0.5  - Remove Singlets: True  - Min. # Scans per Peak: 5  - Min. # Isotopes: 1 |
| Group Compounds: | 1. Compound Consolidation:  - Mass Tolerance: 5 ppm  - RT Tolerance [min]: 0.2  2. Fragment Data Selection:  - Preferred Ions: [M+H]+1 |
| Search mzCloud: | Search Settings:  - Compound Classes: All  - Match Ion Activation Type: True  - Match Ion Activation Energy: Match with Tolerance  - Ion Activation Energy Tolerance: 20  - Apply Intensity Threshold: True  - Precursor Mass Tolerance: 10 ppm  - FT Fragment Mass Tolerance: 10 ppm  - IT Fragment Mass Tolerance: 0.4 Da  - Identity Search: HighChem HighRes  - Similarity Search: Similarity Forward  - Library: Reference  - Post Processing: Recalibrated  - Match Factor Threshold: 50  - Max. # Results: 10 |
| Assign Compound Annotations | General Settings:  - Mass Tolerance: 5 ppm  2. Data Sources:  - Data Source #1: mzCloud Search  - Data Source #2: Predicted Compositions  - Data Source #3: MassList Search  - Data Source #4: ChemSpider Search  - Data Source #5: Metabolika Search |
| Search ChemSpider | Database(s): BioCyc; Human Metabolome Database; KEGG  - Search Mode: By Formula or Mass  - Mass Tolerance: 5 ppm  - Max. # of results per compound: 100  - Max. # of Predicted Compositions to be searched per Compound: 3  - Result Order (for Max. # of results per compound): Order By Reference Count (DESC)  2. Predicted Composition Annotation:  - Check All Predicted Compositions: False |
| Apply mzLogic | 1. Search Settings:  - FT Fragment Mass Tolerance: 10 ppm  - IT Fragment Mass Tolerance: 0.4 Da  - Max. # Compounds: 0  - Max. # mzCloud Similarity Results to consider per Compound: 10  - Match Factor Threshold: 30 |
